# Supplementary figures and images for: Pramipexole has a neuroprotective effect in spinal cord injury and upregulates D2 receptor expression in the injured spinal cord tissue in rats
Source: PeerJ. 2023 Sep 11;11:e16039. doi: 10.7717/peerj.16039 (PMC10501368; doi:10.7717/peerj.16039)

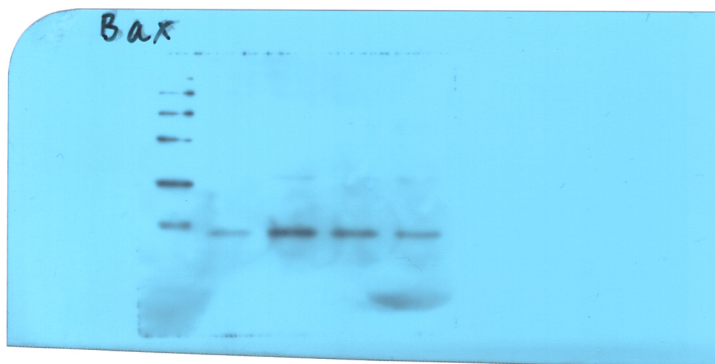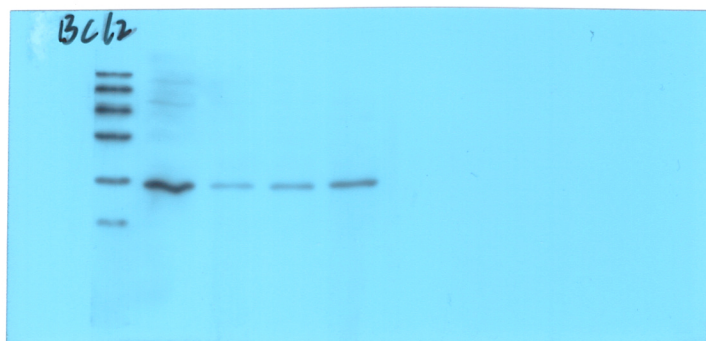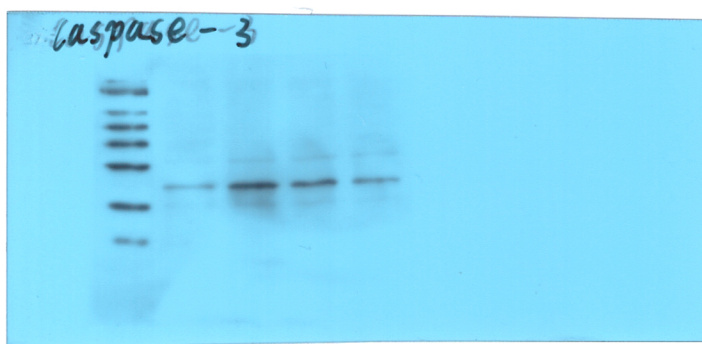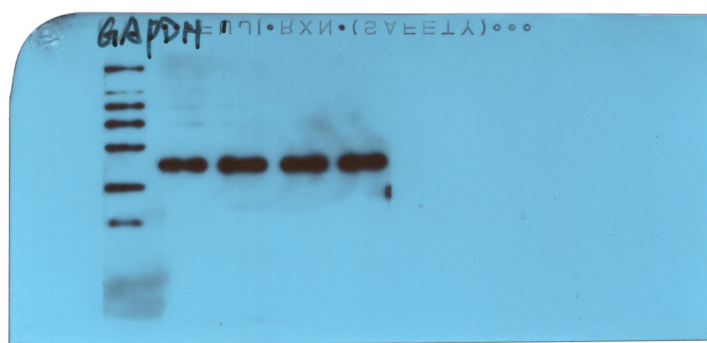

Supplement: Supplemental Information 1 [file peerj-11-16039-s001.zip › WB blots/Figure 2A bax-bcl.pdf]

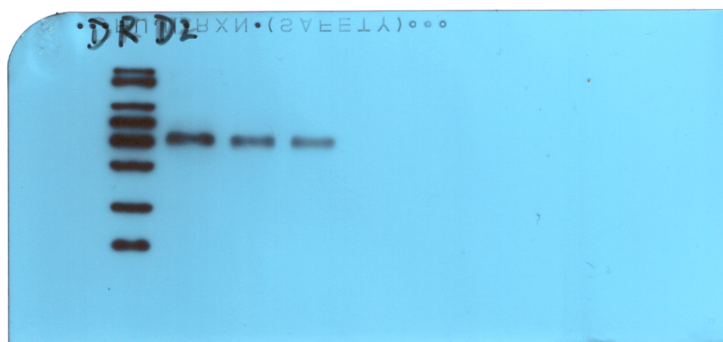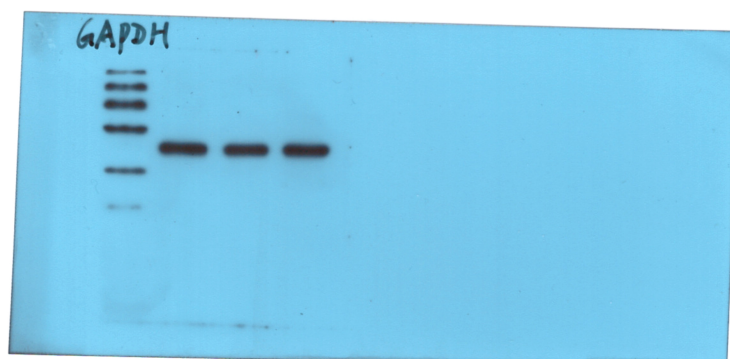

Supplement: Supplemental Information 1 [file peerj-11-16039-s001.zip › WB blots/Figure 4A DRD2.pdf]

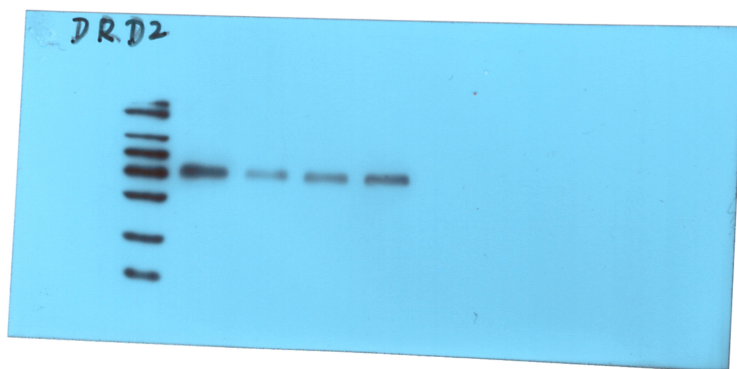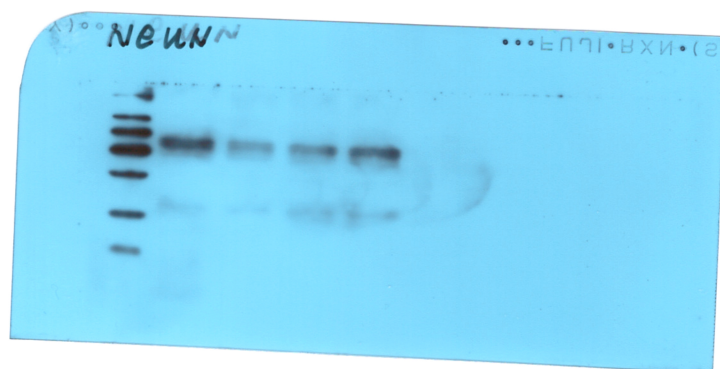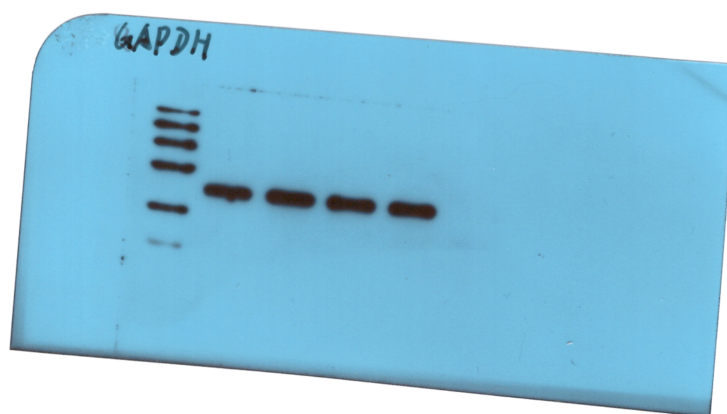

Supplement: Supplemental Information 1 [file peerj-11-16039-s001.zip › WB blots/Figure 4C DRD2-Neun.pdf]

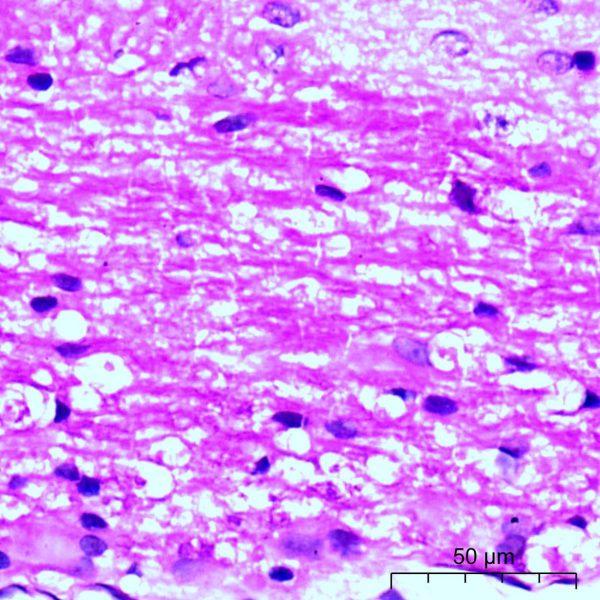

Supplement: Supplemental Information 2 [file peerj-11-16039-s002.zip › Fig 1/fig 1D/Sham.png]

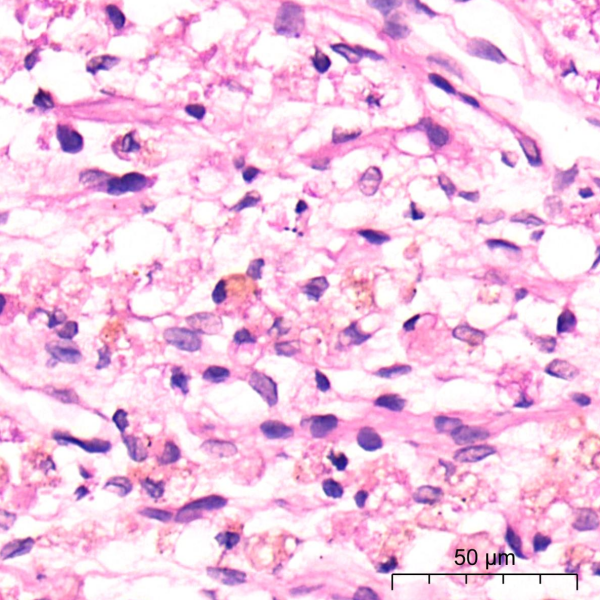

Supplement: Supplemental Information 2 [file peerj-11-16039-s002.zip › Fig 1/fig 1D/PPX-0.25.png]

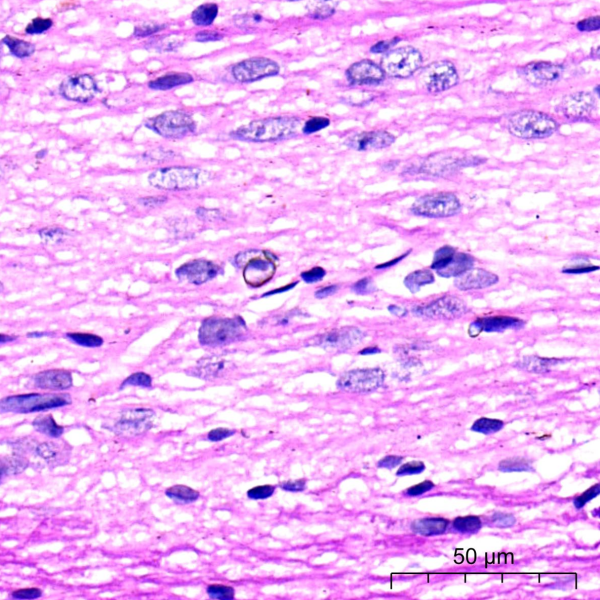

Supplement: Supplemental Information 2 [file peerj-11-16039-s002.zip › Fig 1/fig 1D/PPX-2.0.png]

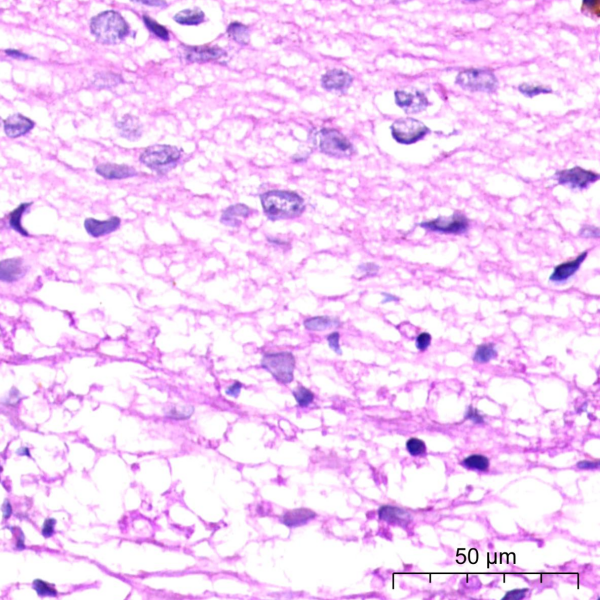

Supplement: Supplemental Information 2 [file peerj-11-16039-s002.zip › Fig 1/fig 1D/SCI.png]

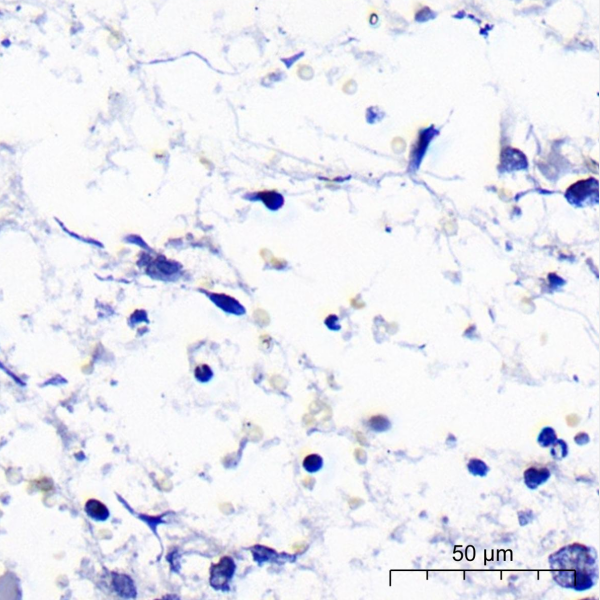

Supplement: Supplemental Information 2 [file peerj-11-16039-s002.zip › Fig 1/fig 1E/PP-0.25.png]

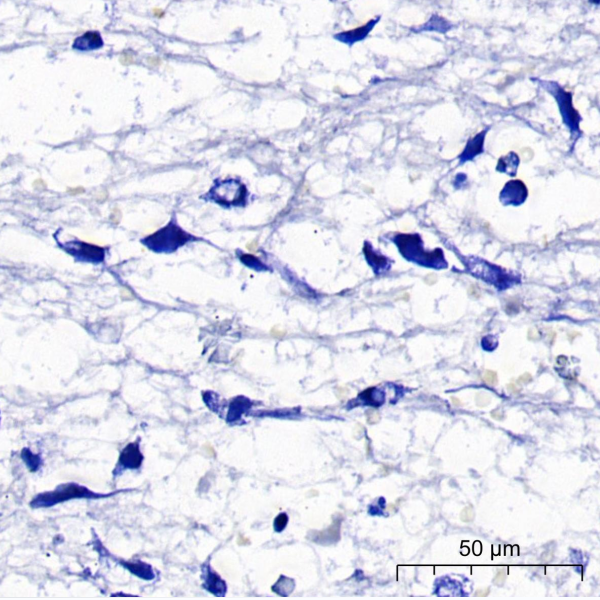

Supplement: Supplemental Information 2 [file peerj-11-16039-s002.zip › Fig 1/fig 1E/PP-2.0.png]

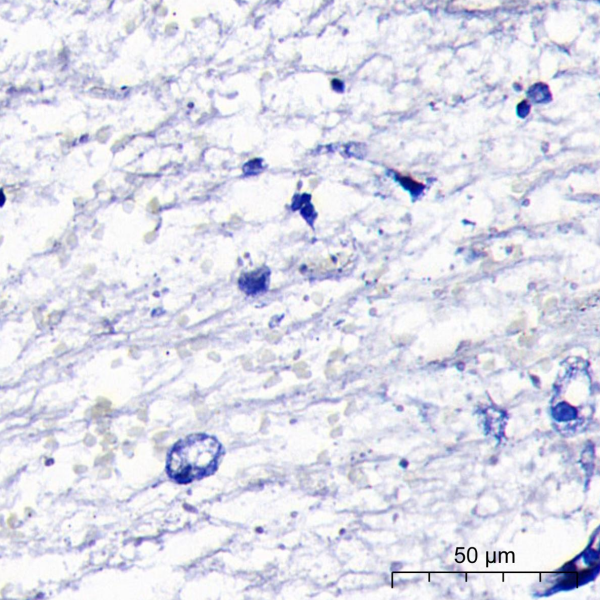

Supplement: Supplemental Information 2 [file peerj-11-16039-s002.zip › Fig 1/fig 1E/SCI 7.png]

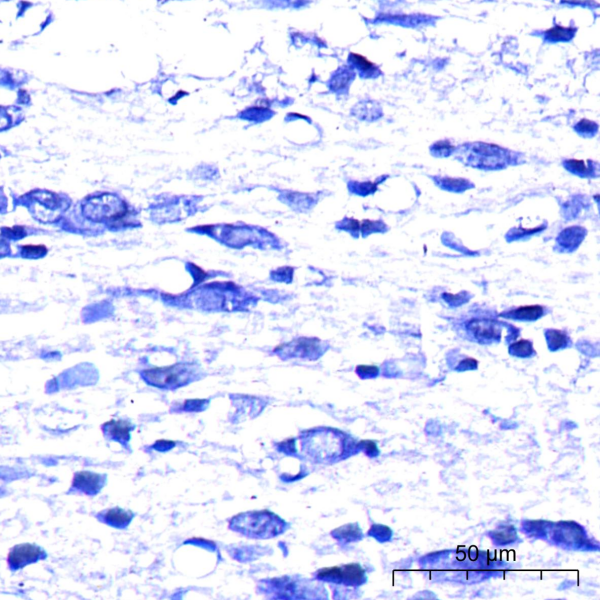

Supplement: Supplemental Information 2 [file peerj-11-16039-s002.zip › Fig 1/fig 1E/Sham 2.png]

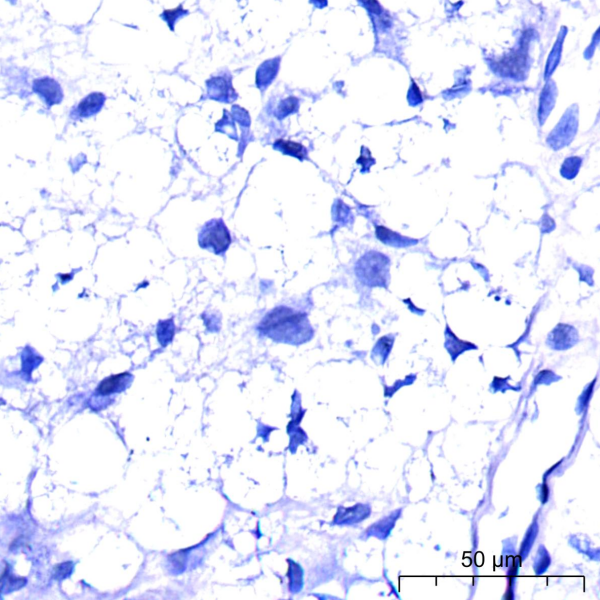

Supplement: Supplemental Information 2 [file peerj-11-16039-s002.zip › Fig 1/fig 1G/PPX-0.25 8.png]

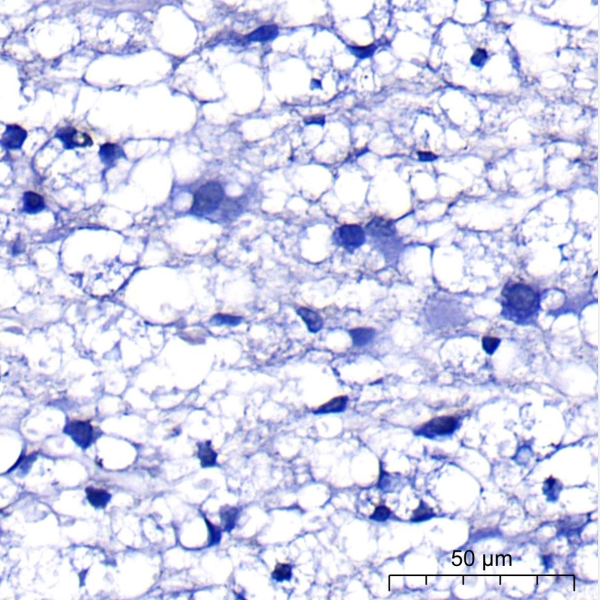

Supplement: Supplemental Information 2 [file peerj-11-16039-s002.zip › Fig 1/fig 1G/PPX-2.0 12.png]

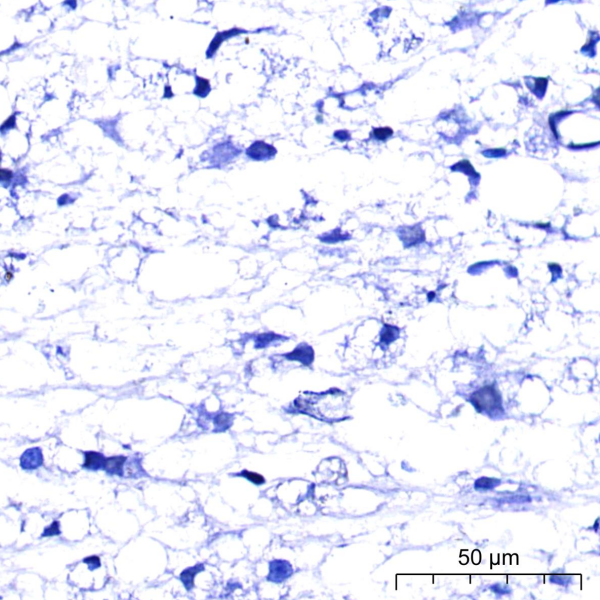

Supplement: Supplemental Information 2 [file peerj-11-16039-s002.zip › Fig 1/fig 1G/SCI 5.png]

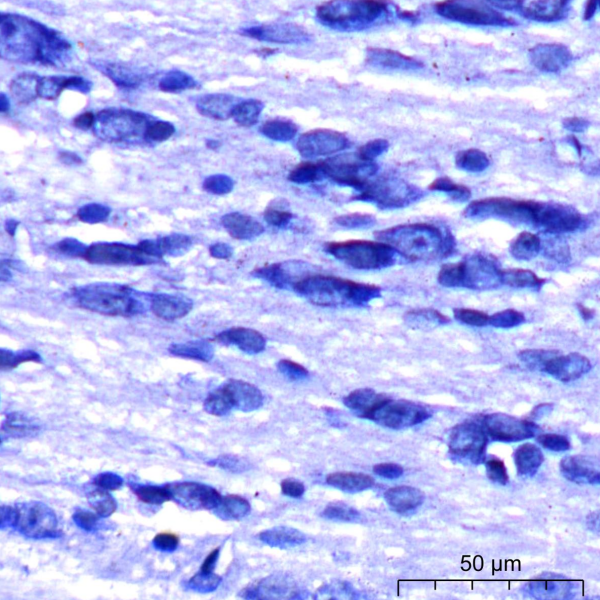

Supplement: Supplemental Information 2 [file peerj-11-16039-s002.zip › Fig 1/fig 1G/Sham 5.png]

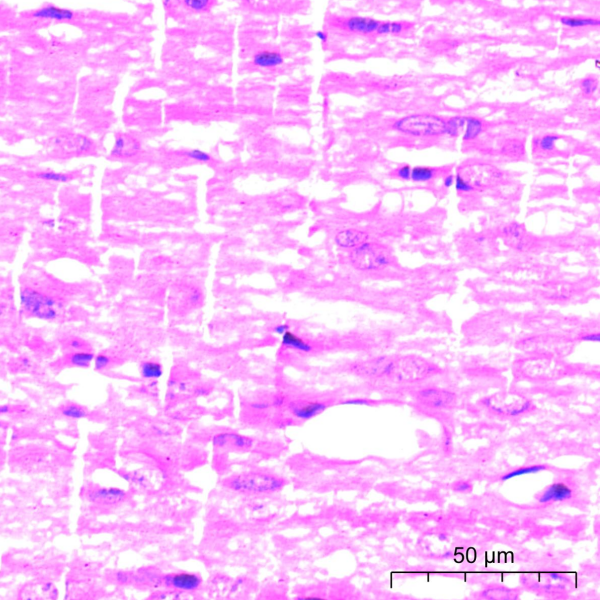

Supplement: Supplemental Information 2 [file peerj-11-16039-s002.zip › Fig 1/fig 1C/PPX-0.25.png]

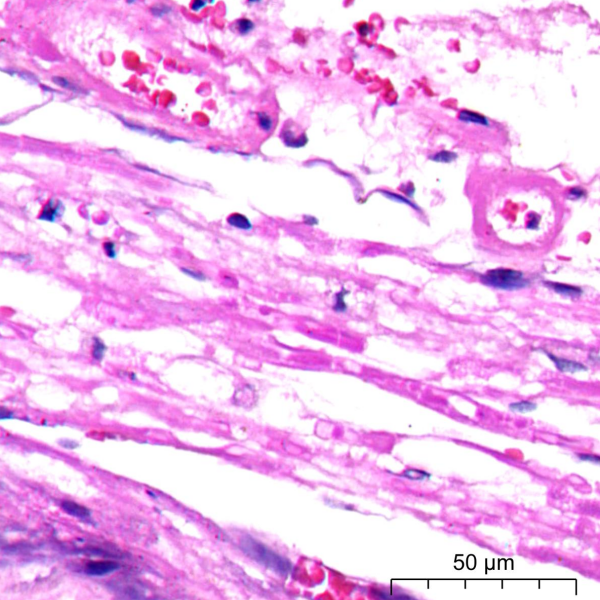

Supplement: Supplemental Information 2 [file peerj-11-16039-s002.zip › Fig 1/fig 1C/PPX-2.0.png]

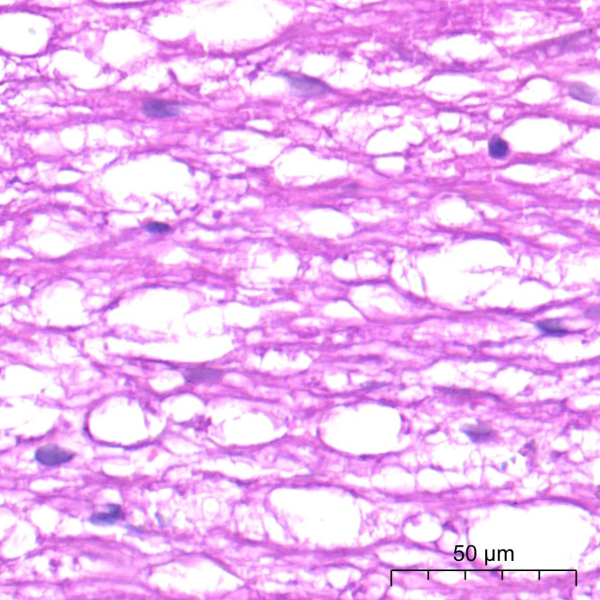

Supplement: Supplemental Information 2 [file peerj-11-16039-s002.zip › Fig 1/fig 1C/SCI.png]

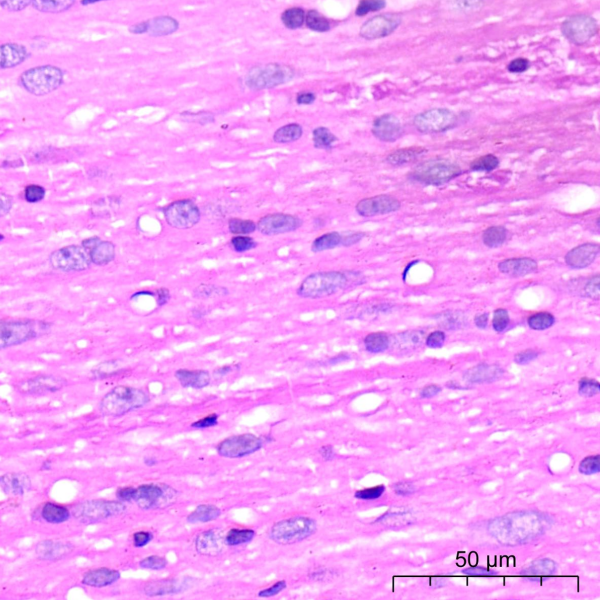

Supplement: Supplemental Information 2 [file peerj-11-16039-s002.zip › Fig 1/fig 1C/Sham.png]

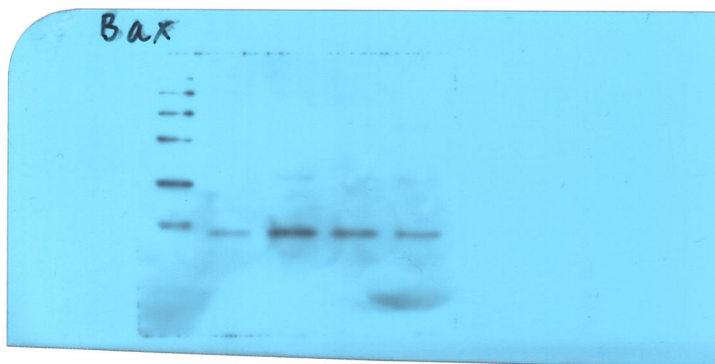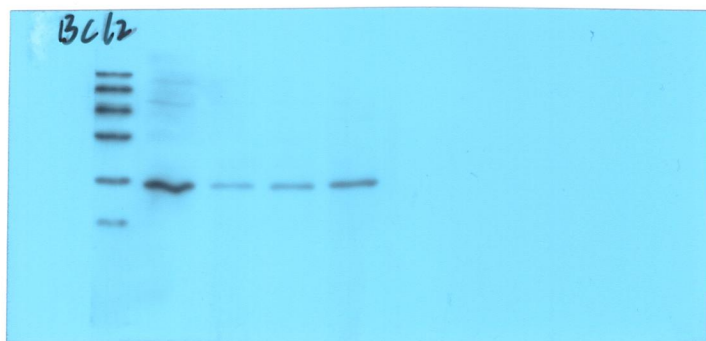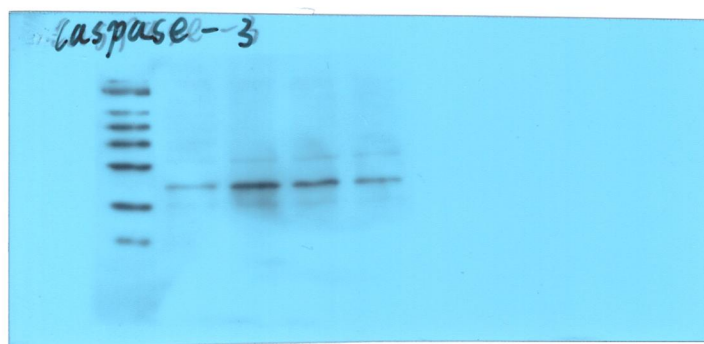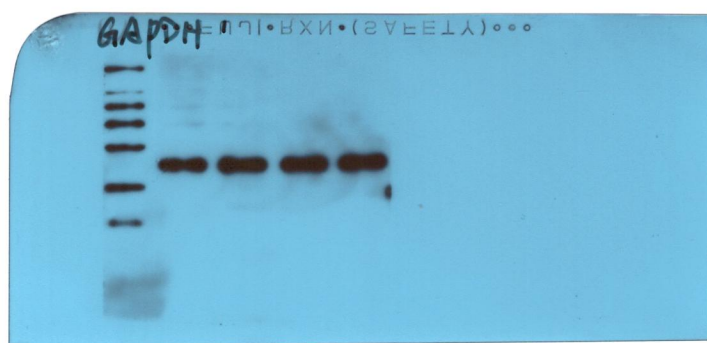

Supplement: Supplemental Information 2 [file peerj-11-16039-s002.zip › Fig 2/fig 2A/bax-bcl.pdf]

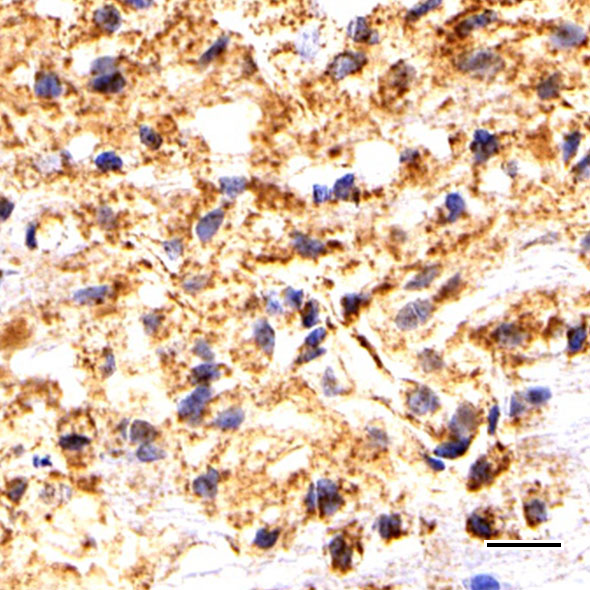

Supplement: Supplemental Information 2 [file peerj-11-16039-s002.zip › Fig 2/fig 2FH/3 d PPX-0.25.tif]

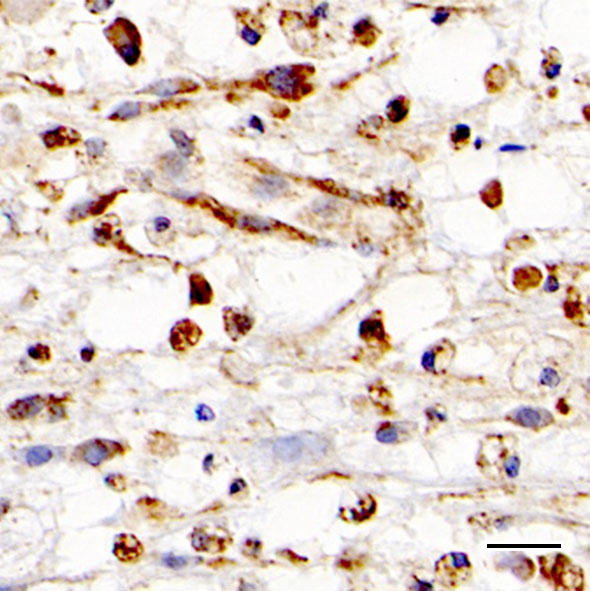

Supplement: Supplemental Information 2 [file peerj-11-16039-s002.zip › Fig 2/fig 2FH/3 d PPX-2.0.tif]

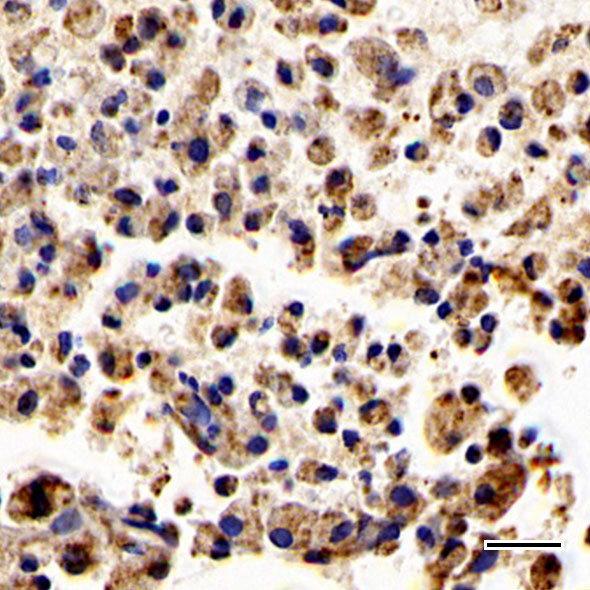

Supplement: Supplemental Information 2 [file peerj-11-16039-s002.zip › Fig 2/fig 2FH/3 d SCI 1.tif]

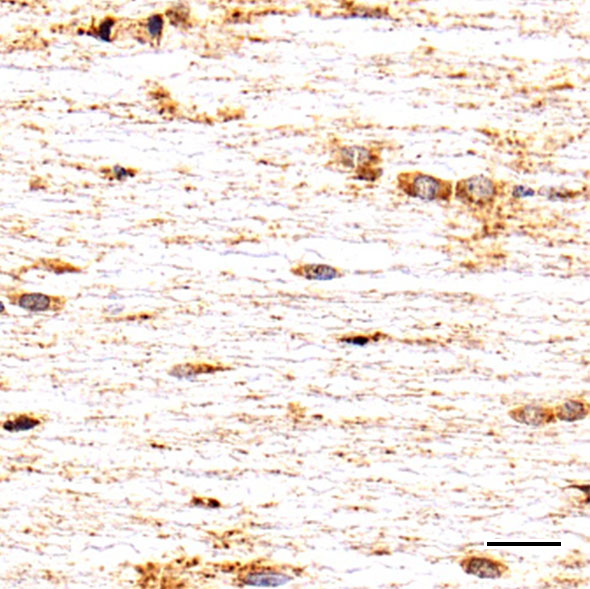

Supplement: Supplemental Information 2 [file peerj-11-16039-s002.zip › Fig 2/fig 2FH/3 d Sham 3.tif]

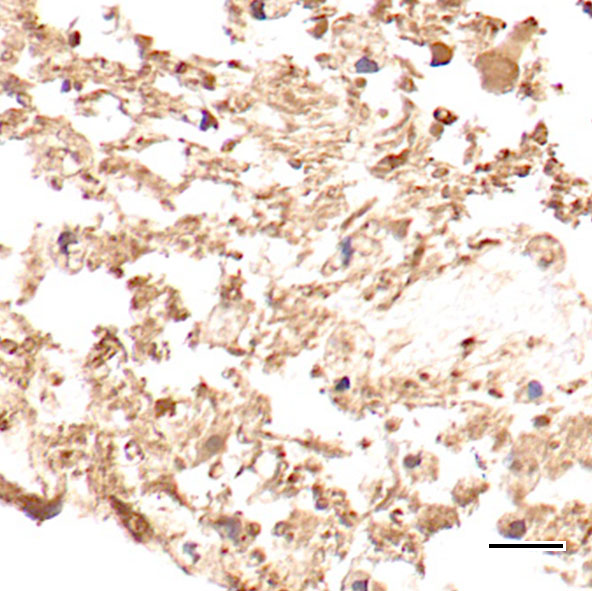

Supplement: Supplemental Information 2 [file peerj-11-16039-s002.zip › Fig 2/fig 2GI/3d SCI.tif]

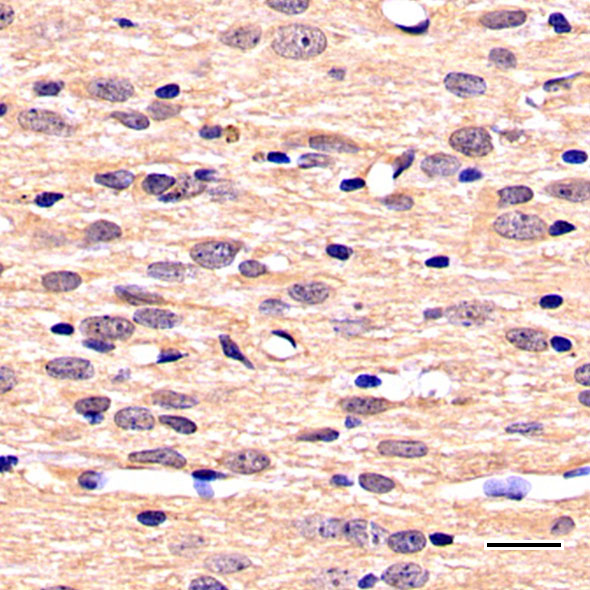

Supplement: Supplemental Information 2 [file peerj-11-16039-s002.zip › Fig 2/fig 2GI/3d Sham.tif]

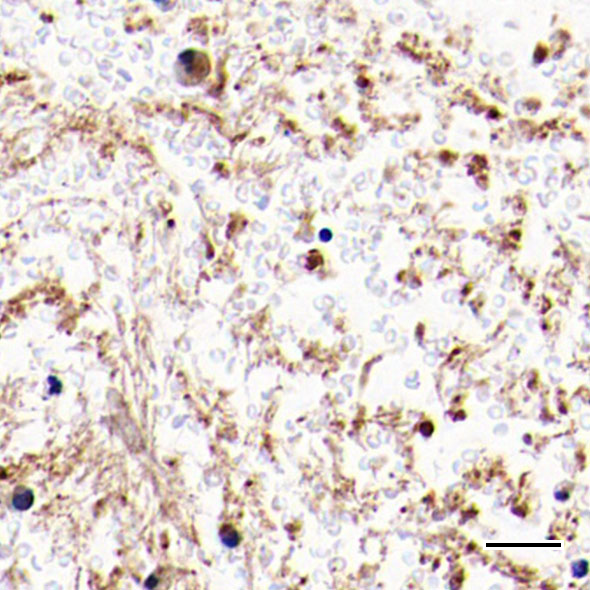

Supplement: Supplemental Information 2 [file peerj-11-16039-s002.zip › Fig 2/fig 2GI/3d PPX-0.25.tif]

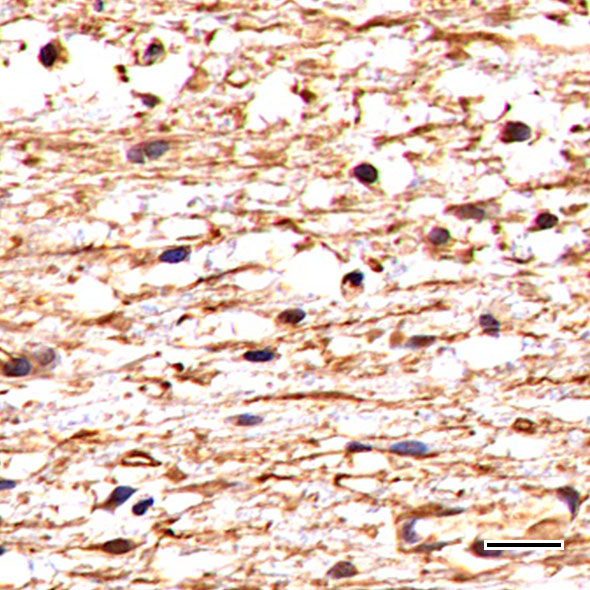

Supplement: Supplemental Information 2 [file peerj-11-16039-s002.zip › Fig 2/fig 2GI/3d PPX-2.0.tif]

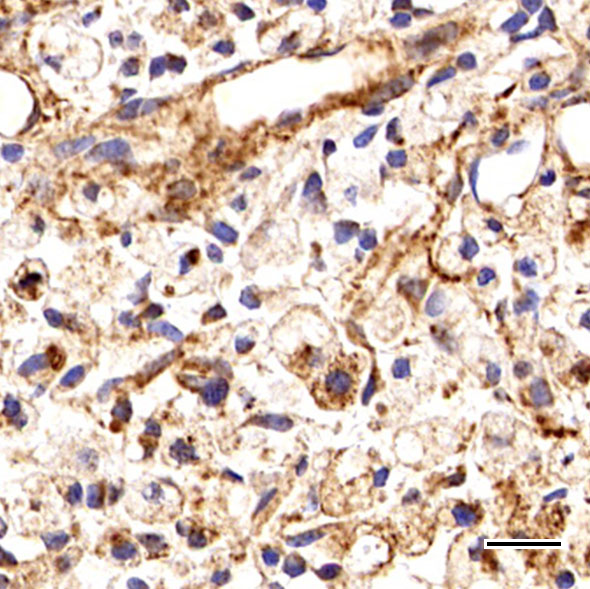

Supplement: Supplemental Information 2 [file peerj-11-16039-s002.zip › Fig 2/fig 2JL/7d SCI.tif]

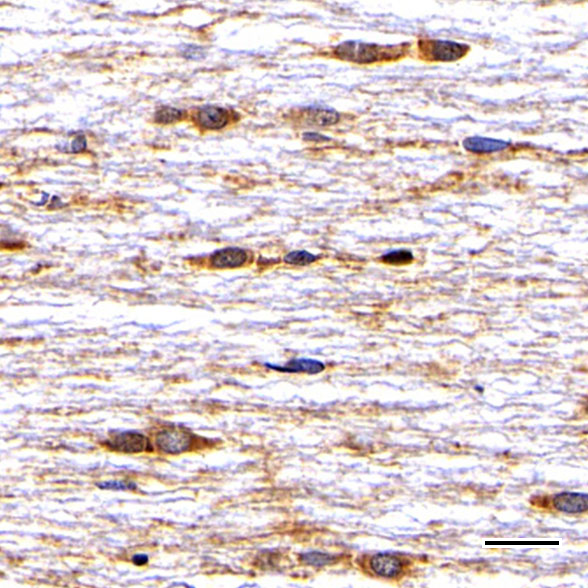

Supplement: Supplemental Information 2 [file peerj-11-16039-s002.zip › Fig 2/fig 2JL/7d Sham.tif]

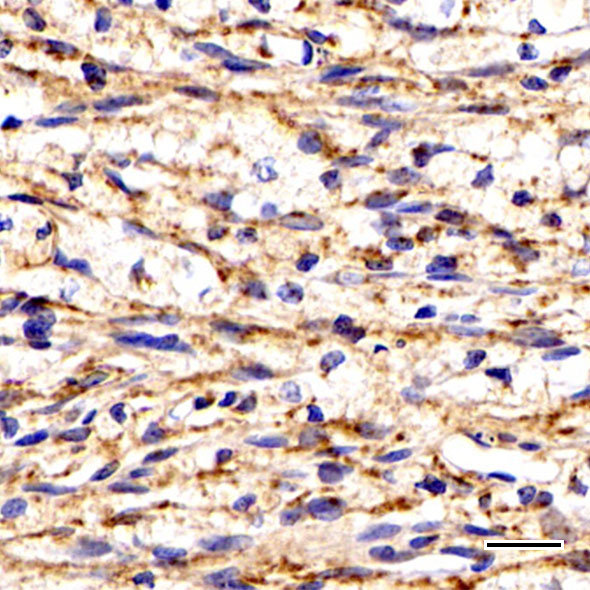

Supplement: Supplemental Information 2 [file peerj-11-16039-s002.zip › Fig 2/fig 2JL/7d PPX-0.25.tif]

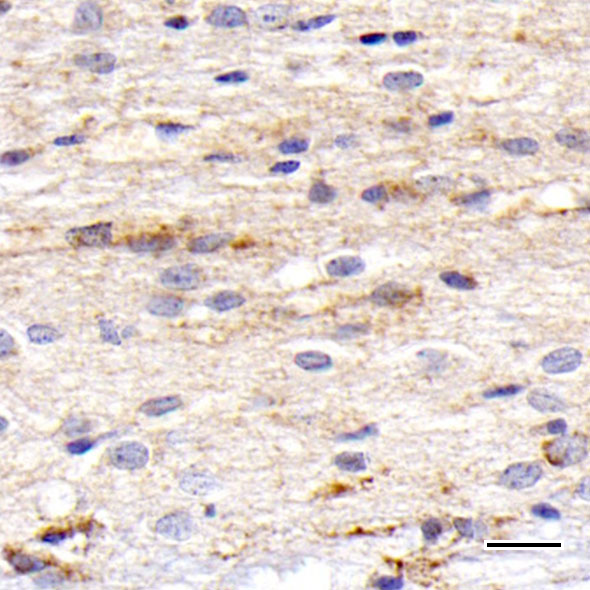

Supplement: Supplemental Information 2 [file peerj-11-16039-s002.zip › Fig 2/fig 2JL/7d PPX-2.0.tif]

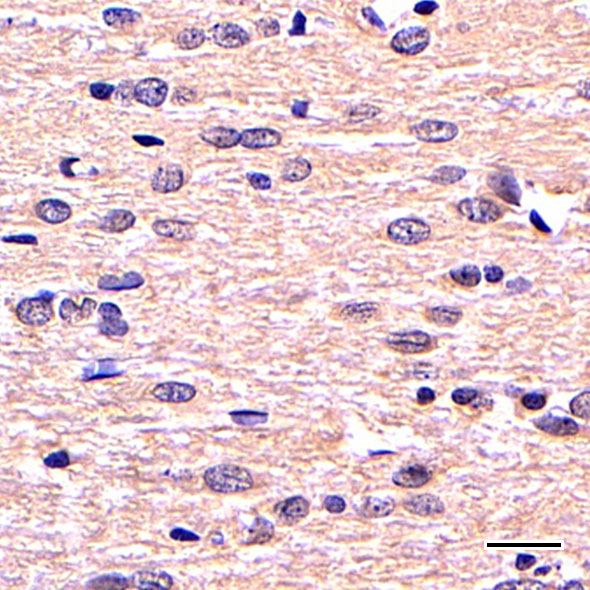

Supplement: Supplemental Information 2 [file peerj-11-16039-s002.zip › Fig 2/fig 2KM/Sham.tif]

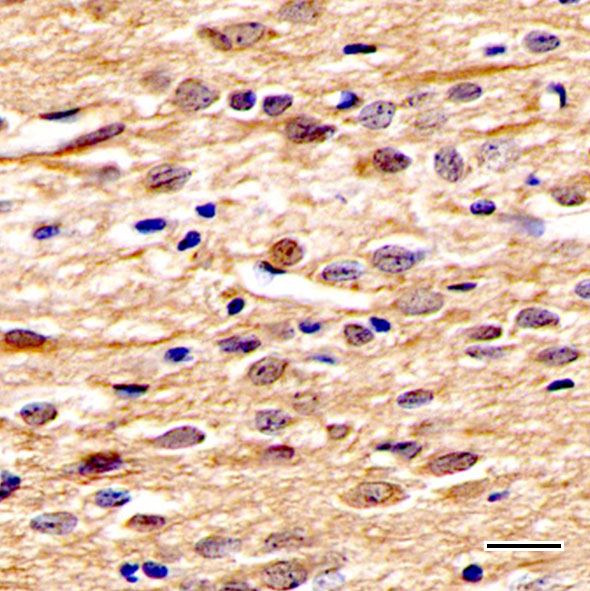

Supplement: Supplemental Information 2 [file peerj-11-16039-s002.zip › Fig 2/fig 2KM/PPX-2.0.tif]

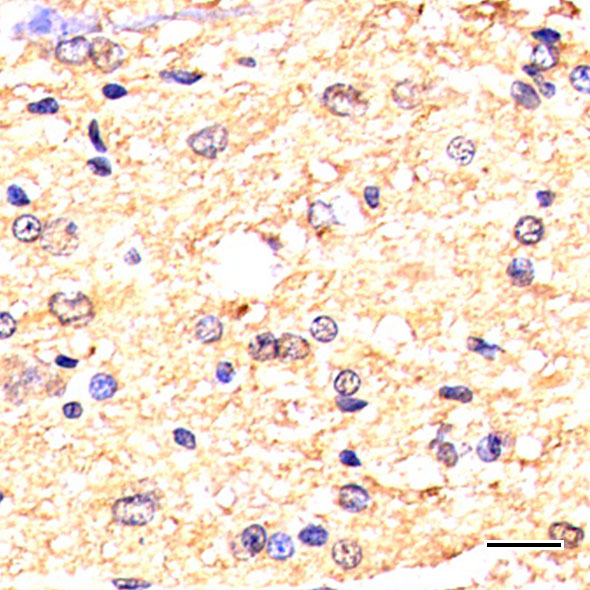

Supplement: Supplemental Information 2 [file peerj-11-16039-s002.zip › Fig 2/fig 2KM/SCI.tif]

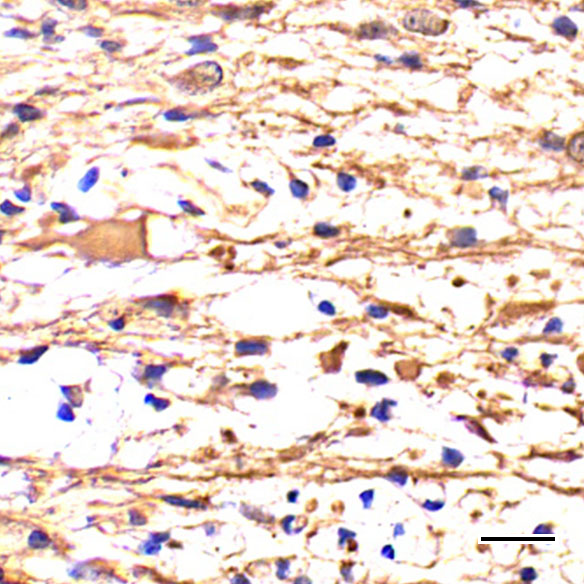

Supplement: Supplemental Information 2 [file peerj-11-16039-s002.zip › Fig 2/fig 2KM/PPX-0.25.tif]

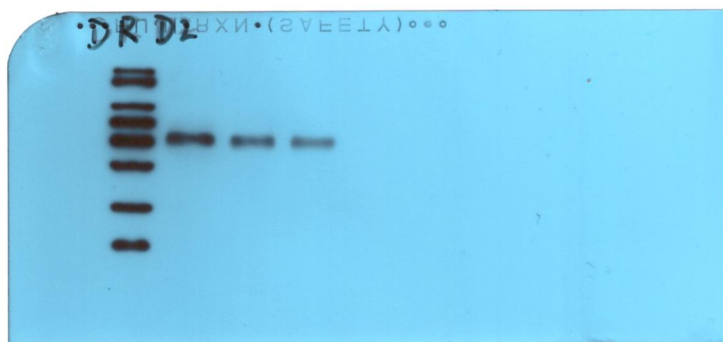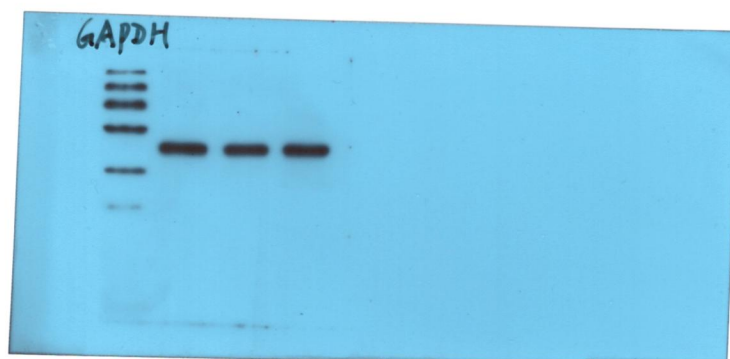

Supplement: Supplemental Information 2 [file peerj-11-16039-s002.zip › Fig 4/fig 4A/DRD2.pdf]

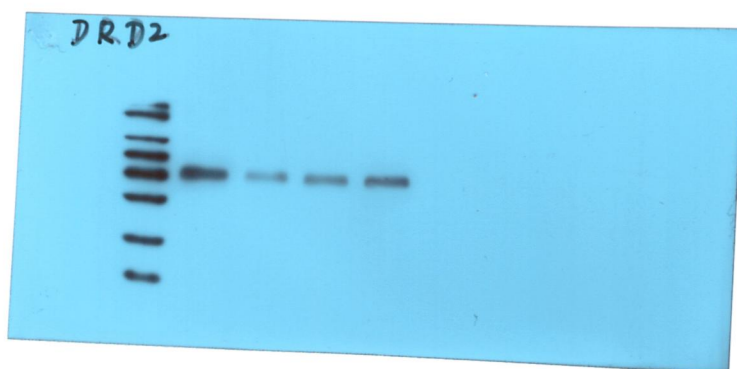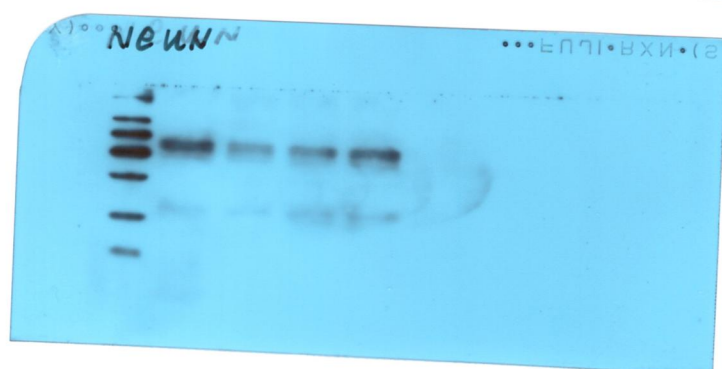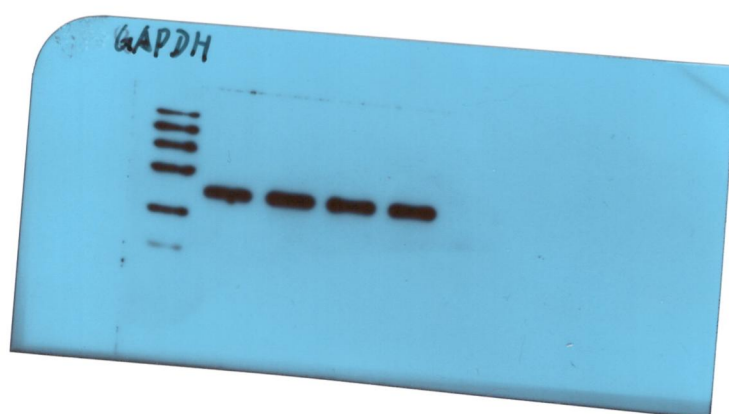

Supplement: Supplemental Information 2 [file peerj-11-16039-s002.zip › Fig 4/fig 4C/DRD2-Neun.pdf]
